# Outcomes of Time to Treatment With Reteplase for Acute Ischemic Stroke: The RAISE Trial Subgroup

**DOI:** 10.1016/j.jacasi.2024.12.010

**Published:** 2025-02-11

**Authors:** Xuechun Wang, Baoyu Feng, Hong-Qiu Gu, Zixiao Li, Yilong Wang, Xingquan Zhao, Shuya Li, Yongjun Wang

**Affiliations:** a Department of Neurology, Beijing Tiantan Hospital, Capital Medical University, Beijing, China; b Department of Clinical Trial Center, Beijing Tiantan Hospital, Capital Medical University, Beijing, China; c China National Clinical Research Center for Neurological Diseases, Capital Medical University, Beijing, China

**Keywords:** alteplase, intravenous thrombolysis, onset-to-treatment time, reteplase, stroke

## Abstract

**Background:**

The efficacy of intravenous thrombolysis is time-dependent. Reteplase has been shown to be superior to alteplase in certain acute ischemic stroke patients.

**Objectives:**

The authors aimed to delineate the associations of stroke onset-to-treatment time (OTT) on the therapeutic benefits and clinical risks with reteplase in comparison to alteplase.

**Methods:**

This is a post hoc analysis of the RAISE (Reteplase versus Alteplase for Acute Ischemic Stroke) trial. Patients were divided into 3 groups based on their onset-to-treatment times: 0 to 90, 91 to 180, and 181 to 270 minutes. The primary efficacy outcome was the proportion of participants with a modified Rankin scale score of 0 to 1 at 90 days. The primary safety outcome was symptomatic intracranial hemorrhage within 36 hours post–thrombolytic treatment.

**Results:**

A total of 1,399 patients (99.1%) with OTT (median 180 minutes; Q1-Q3: 135-222 minutes) were included. Adjusted risk ratios of primary efficacy outcome were 1.16 (95% CI: 0.70-1.91) for the 0 to 90 minutes group, 1.14 (95% CI: 0.97-1.35) for 91 to 180 minutes group, and 1.12 (95% CI: 0.93-1.18) for 181 to 270 minutes group. The primary safety outcome had no difference between reteplase and alteplase in the 3 OTT intervals.

**Conclusions:**

Among patients with ischemic stroke within 4.5 hours after symptom onset, there was no significant difference in the efficacy profile between reteplase and alteplase for achieving excellent functional outcomes at 3 different OTT intervals. (A Study of r-PA Treating Patients With Acute Ischemic Stroke [RAISE]; NCT05295173)

Intravenous alteplase is recommended by guidelines within 4.5 hours after the symptoms onset of acute ischemic stroke (AIS).^1^, ^2^, ^3^ Reteplase as a third-generation thrombolytic agent derived from human tissue plasminogen activator, and is administered through 2 separate intravenous injections, with a time interval of 30 minutes. Unlike some other thrombolytic agents that may have weight-based dosing, reteplase is given in fixed doses.^4^, ^5^, ^6^, ^7^, ^8^ This streamlines the administrative process, facilitating efficient management during emergency situations. Reteplase has been approved for acute myocardial infarction treatment in numerous regions,^4^^,^^6^^,^^7^^,^^9^^,^^10^ and demonstrated significant promise as a potential therapeutic agent for stroke treatment.^11^^,^^12^

The key to managing AIS lies in promptly reestablishing blood flow in the occluded vessels to salvage the ischemic penumbra. Two meta-analyses confirm significant benefits of intravenous rt-PA administered within 4.5 hours of stroke symptoms, with earlier treatment yielding greater benefits.^13^^,^^14^ Given the practical and efficient advantages of reteplase over alteplase, there exists a hypothesis suggesting that reteplase might yield more excellent functional outcomes at the 90-day mark in patients with ischemic stroke who present to the emergency department at a late time window. However, this potential benefit needs to be carefully balanced against the increased risk of hemorrhagic complications.

In the RAISE (Reteplase versus Alteplase for Acute Ischemic Stroke) trial, reteplase was proved to be more likely to result in an excellent functional outcome than alteplase among patients with ischemic stroke within 4.5 hours after symptom onset.^11^ In this post hoc analysis of the RAISE trial, we aimed to delineate the therapeutic benefit and clinical risks of reteplase compared with alteplase within 3 different onset-to-treatment time (OTT) intervals (0-90, 91-180, and 181-270 minutes).

## Methods

### Overview of the RAISE trial

The RAISE trial was a phase 3, multicenter, prospective, open-label, noninferiority, randomized trial with blinded endpoint assessment conducted across 62 Chinese sites. The trial aimed to evaluate the efficacy of a double-bolus 18 mg plus 18 mg dose of reteplase against standard alteplase, focusing on functional outcomes in patients with AIS treated with intravenous thrombolysis within 4.5 hours of symptom onset. The study protocol, statistical analysis plan and the primary results of the trial have been published previously.^11^^,^^15^ The study protocol was granted approval by the Institutional Review Boards across all study sites, and written informed consent was obtained from all patients before their involvement.

Eligibility criteria for the study included patients ages 18 to 80 years, capable of receiving intravenous thrombolysis within 4.5 hours of their last known well state, with a prestroke functional status indicated by a modified Rankin scale (mRS) score of ≤1 (0 indicating no deficit to 6 for death), and a disabling ischemic stroke with a National Institutes of Health Stroke Scale (NIHSS) score ranging from 4 to 25 (range 0 [no neurologic deficit] to 42 [death]).

Patients were randomly assigned in a 1:1 ratio to receive either intravenous reteplase or alteplase. Reteplase was administered as 2 18-mg bolus doses, infused over 2 minutes each, with a 30-minute interval between doses. Alteplase was given at 0.9 mg/kg (up to 90 mg), with an initial 10% bolus over 1 minute, followed by a 60-minute infusion. Treatment was open-label, and all other care adhered to standard ischemic stroke management protocols.

The primary efficacy outcome was the proportion of excellent functional outcome defined as an mRS score of 0 to 1 at 90 days. The primary safety outcomes were the rate of symptomatic intracranial hemorrhage within 36 hours, as defined by the European Cooperative Acute Stroke Study III.^16^ The primary efficacy and safety outcomes of this post hoc subgroup analysis aligned with those of the overarching RAISE trial analysis. Early dramatic recovery on the NIHSS score was defined as a decrease of at least 4 points or a score no more than 1. Additional safety outcomes included symptomatic intracranial hemorrhage within 7 days, as per the ECASS III (European Cooperative Acute Stroke Study III) criteria^16^; parenchymal hemorrhage 2 intracranial hemorrhage within 36 hours, defined by the SITS-MOST (Safe Implementation of Thrombolysis in Stroke-Monitoring Study)^17^; any intracranial hemorrhage; and hemorrhagic events categorized as major or clinically relevant nonmassive, in accordance with the International Society on Thrombosis and Haemostasis guidelines.^18^^,^^19^

### Subgroup analysis

In this analysis, we performed a post hoc subgroup examination of the RAISE trial, evaluating efficacy and safety outcomes across 3 defined OTT intervals: 0 to 90, 91 to 180, and 181 to 270 minutes, consistent with criteria utilized in prior clinical trials.^20^, ^21^, ^22^, ^23^

Between March 21, 2022, and June 22, 2023, 1,412 patients with AIS in 62 study sites in China were enrolled in this randomized study and comprised the intention-to-treat population. In total, 707 patients were designated to receive treatment with reteplase and 705 were with alteplase (Supplemental Table 1). In this subgroup analysis, 7 patients in the reteplase group and 6 patients in the alteplase group who did not receive the assigned treatment were excluded. Finally, a total of 1,399 patients (99.1%) with OTT were included.

### Statistical analysis

As an exploratory analysis, we conducted the analysis in the complete data. Categorical variables were described as cases and proportions, and continuous variables were described by mean ± SD or median (IQR) range. For difference among groups by OTT time, chi-square test, Fisher exact test, analysis of variance test, or Kruskal-Wallis H test was used; for difference between treatment and control, chi-square test, Fisher exact test, Student’s *t*-test, or Mann-Whitney *U* test was used.

We performed analysis in the modified intention-to-treat population. For primary efficacy outcome, univariate and multivariable robust Poisson regression models with trial center as a random effect were used to calculate the risk ratio (RR) and related 95% CI of the treatment group compared with the control group. Interaction of treatment and OTT were also estimated in the model. To control potential bias, in the multivariable model, age, sex, weight, pre-mRS, NIHSS at baseline, and comorbidities (including hypertension, diabetes, hyperlipidemia, coronary heart disease, and arrhythmia) were adjusted. The adjusted analysis was the primary analysis in the subgroup analysis. An interaction between OTT by treatment interaction treating time as a continuum was tested. We further conducted stratified analysis by age, sex, weight, NIHSS score at admission, mRS score, and comorbidities (including hypertension, diabetes, hyperlipidemia, coronary heart disease, and arrhythmia). For dichotomous secondary efficacy outcomes and safety outcomes, a similar analysis strategy was used. Ordinal logistic regression for the ordinal mRS score was conducted with common OR and 95% CI reported.

All statistical analyses were performed by SAS software, version 9.4 (SAS Institute Inc). All *P* values were reported as 2-sided, and *P <* 0.05 was set to be statistically significant.

## Results

### Baseline characteristics

A total of 1,399 participants were included in the study with median OTT of 180 minutes (Q1-Q3: 135 to 222 minutes). There were 91 (6.5%) patients with OTT of 0 to 90 minutes (median 76.0 minutes; Q1-Q3: 62.0-87.0minutes), 612 (43.7%) patients with OTT of 91 to 180 minutes (median 141.0 minutes; Q1-Q3: 119.0-162.0 minutes), and 696 (49.7%) patients with OTT of 181 to 270 minutes (median 222.0 minutes; Q1-Q3: 200.0-244.0 minutes). Baseline characteristics of patients grouped by OTT were shown in Supplemental Table 1. In the 0 to 90 minutes interval, patients exhibited a younger age *(P =* 0.006), higher male predominance *(P =* 0.005), increased weight *(P =* 0.040), greater prevalence of hyperlipidemia *(P =* 0.002), and a higher proportion of patients with NIHSS score group exceeding 7 points at admission *(P =* 0.040). Moreover, there was a shorter symptom onset-to-treatment time (*P <* 0.001) and door to treatment time (*P <* 0.001). Additionally, fewer bridging thrombectomy procedures were performed *(P =* 0.040). Notably, no significant differences were observed in hypertension, diabetes, coronary heart disease, arrhythmia, or mRS score before stroke in the 3 groups.

The distribution of demographic and clinical characteristics across the treatment groups demonstrated balance, with no significant differences observed in gender, weight, hypertension, diabetes, hyperlipidemia, coronary heart disease, the door to treatment time, and bridging thrombectomy (Table 1). In the OTT of 91 to 180 minutes interval, the number of patients aged over 60 years was higher in the reteplase group (n = 200, 64.1%) than that in the alteplase group (n = 163, 54.3%; *P =* 0.01). Additionally, the severity of strokes, as indicated by the NIHSS, was greater in the reteplase group (7.0; Q1-Q3: 5.0-9.0), compared to the alteplase group's (6.0; Q1-Q3: 5.0-8.0; *P =* 0.04). In the OTT of 181 to 270 minutes interval, fewer patients had a pre-mRS score of 1 to 2 in the reteplase group (16, 4.7%) than in the alteplase group (32, 9.1%; *P =* 0.02).

**Table 1:** Baseline Characteristics in the Modified Intention-to-Treat Population Grouped by Onset-to-Treatment Time and Treatments

	Onset-to-Treatment Time of 0-90 min			Onset-to-Treatment Time of 91-180 min			Onset-to-Treatment Time of 181-270 min		
	Reteplase (n = 45 [49.5%])	Alteplase (n = 46 [50.2%])	*P* Value	Reteplase (n = 312 [51.0%])	Alteplase (n = 300 [49.0%])	*P* Value	Reteplase (n = 343 [49.3%])	Alteplase (n = 353 [50.7%])	*P* Value
Age, y	60.0 (54.0-65.0)	59.0 (53.0-66.0)	0.57	64.0 (57.0-71.0)	62.5 (54.5-69.0)	0.05	63.0 (55.0-70.0)	64.0 (57.0-70.0)	0.38
Age group			0.93			0.01			0.09
18-60 y	25 (55.6)	26 (56.5)		112 (35.9)	137 (45.7)		152 (44.3)	134 (38.0)	
>60 y	20 (44.4)	20 (43.5)		200 (64.1)	163 (54.3)		191 (55.7)	219 (62.0)	
Sex			0.73			0.62			0.25
Female	7 (15.6)	6 (13.0)		92 (29.5)	94 (31.3)		98 (28.6)	115 (32.6)	
Male	38 (84.4)	40 (87.0)		220 (70.5)	206 (68.7)		245 (71.4)	238 (67.4)	
Weight, kg	71.0 (63.0-78.0)	72.5 (62.0-80.0)	0.71	68.0 (60.0-75.0)	68.2 (60.0-75.0)	0.66	69.0 (60.0-75.0)	66.0 (60.0-75.0)	0.14
Comorbidities									
Hypertension	29 (64.4)	32 (69.6)	0.60	228 (73.1)	232 (77.3)	0.22	268 (78.1)	257 (72.8)	0.10
Diabetes	9 (20.0)	10 (21.7)	0.84	79 (25.3)	71 (23.7)	0.63	98 (28.6)	85 (24.1)	0.18
Hyperlipidemia	26 (57.8)	27 (58.7)	0.93	122 (39.1)	123 (41.0)	0.63	130 (37.9)	143 (40.5)	0.48
Coronary heart disease	14 (31.1)	15 (32.6)	0.88	84 (26.9)	76 (25.3)	0.65	78 (22.7)	91 (25.8)	0.35
Arrhythmia^a^	3 (6.7)	10 (21.7)	0.04	41 (13.1)	49 (16.3)	0.26	48 (14.0)	52 (14.7)	0.78
Prestroke mRS^b^			0.72			0.65			0.02
0	42 (93.3)	42 (91.3)		291 (93.3)	277 (92.3)		327 (95.3)	321 (90.9)	
1 or 2^c^	3 (6.7)	4 (8.7)		21 (6.7)	23 (7.7)		16 (4.7)	32 (9.1)	
NIHSS score at admission^d^	7.0 (5.0-9.0)	6.0 (5.0-9.0)	0.18	7.0 (5.0-9.0)	6.0 (5.0-8.0)	0.04	6.0 (5.0-8.0)	6.0 (5.0-8.0)	0.97
NIHSS score group at admission^d^			0.07			0.33			0.37
4-7	23 (51.1)	32 (69.6)		192 (61.5)	196 (65.3)		243 (70.8)	239 (67.7)	
>7	22 (48.9)	14 (30.4)		120 (38.5)	104 (34.7)		100 (29.2)	114 (32.3)	
Symptom onset-to-treatment time, min^c^	79.0 (70.0-87.0)	68.5 (59.0-80.0)	0.05	138.0 (116.0-163.0)	144.0 (123.0-162.0)	0.12	223.0 (200.0-243.0)	221.0 (201.0-245.0)	0.87
Door to treatment time, min^c^	36.0 (28.0-45.0)	37.0 (30.0-50.0)	0.32	57.0 (37.0-77.0)	57.5 (39.0-76.0)	0.95	65.0 (42.0-92.0)	69.0 (45.0-97.0)	0.27
Bridging thrombectomy	1 (2.2)	1 (2.2)	0.99	13 (4.2)	15 (5.0)	0.62	6 (1.7)	9 (2.5)	0.47

Values are median (Q1-Q3) or n (%).

a Arrhythmia includes sinus bradycardia, sinus tachycardia, atrial flutter, atrial fibrillation, premature beats, supraventricular tachycardia, ventricular tachycardia, bundle-branch block, atrioventricular block, and idioventricular rhythm.

b Scores on the modified Rankin scale (mRS) range from 0 (no neurologic deficit, no symptoms, or completely recovered) to 6 (death).

c One patient in each group had an mRS scale score of 2.

d Scores on the National Institutes of Health Stroke Scale (NIHSS) range from 0 to 42, with higher scores indicating more severe stroke.

### Efficacy outcomes

**Figure 1:**
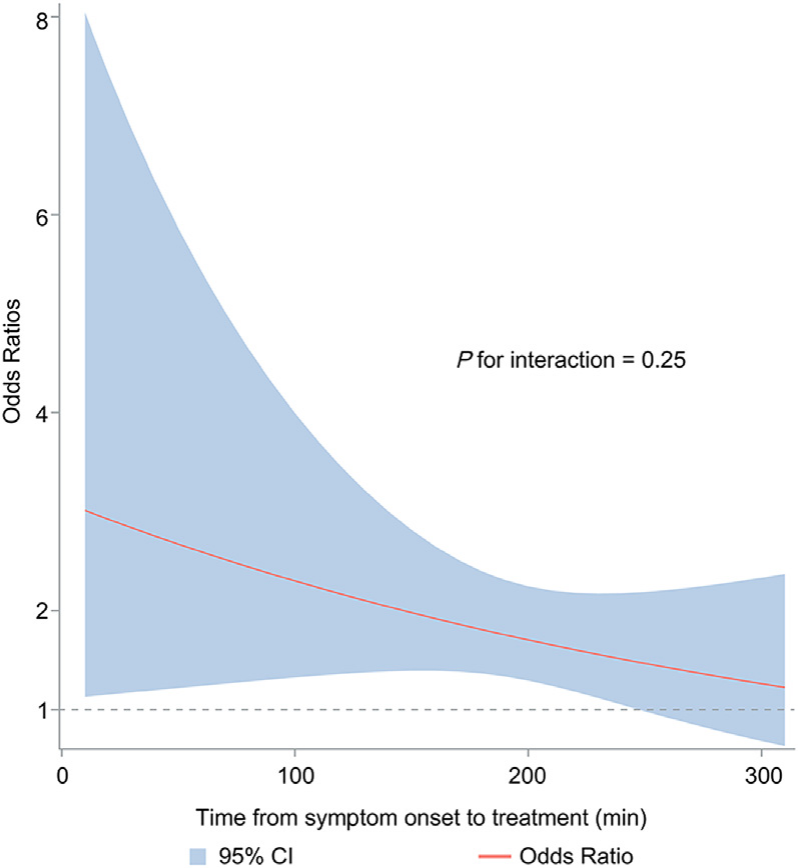
ORs for Modified Rankin Scale Score 0 to 1 of Reteplase vs Alteplase by Onset-to-Treatment Time We used logistic regression to examine the interaction between onset-to-treatment time and treatment, considering time as a continuous variable. The logistic regression model for modified Rankin scale score 0 to 1 revealed a time-dependent trend.

**Figure 2:**
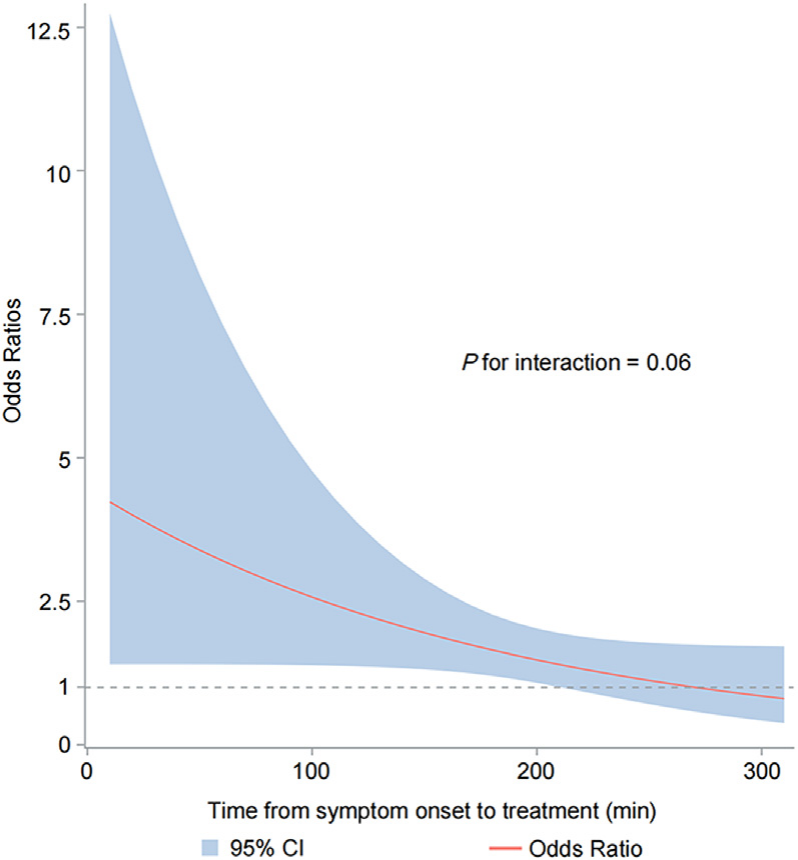
ORs for Modified Rankin Scale Score 0 to 2 of Reteplase vs Alteplase by Onset-to-Treatment Time We used logistic regression to examine the interaction between onset-to-treatment time (OTT) and treatment, considering time as a continuous variable. The logistic regression model for modified Rankin scale score 0 to 2 revealed a time-dependent trend.

Overall, an excellent functional outcome was reported in 80.1% of the patients in the reteplase group and in 71.1% of those in the alteplase group (adjusted RR: 1.13; 95% CI: 1.00-1.28; *P =* 0.050) (Table 2). The adjusted RR of mRS of 0 to 1 at 90 days for the patients with OTT of 0 to 90, 91 to 180, and 181 to 270 minutes was 1.16 (95% CI: 0.70-1.91; *P =* 0.566), 1.14 (95% CI: 0.97-1.35; *P =* 0.117), and 1.12 (95% CI: 0.93-1.34; *P =* 0.220), respectively. No interaction association between OTT and treatment was observed. The subgroup analysis of the primary efficacy outcome was presented in Supplemental Table 2. The adjusted RR of mRS of 0 to 2 at 90 days for the patients with OTT of 0 to 90, 91 to 180, and 181 to 270 minutes was 1.09 (95% CI: 0.69-1.73), 1.11 (95% CI: 0.96-1.28), and 1.03 (95% CI: 0.90-1.19), respectively. Also, no interaction association between OTT and treatment was found. Other secondary efficacy outcomes, which included median mRS at 90 days, early dramatic recovery at 24 hours and 7 days, as well as a BI score ≥95 at 90 days, were detailed in Table 2. The outcomes of the interaction between OTT and treatment, with treating time considered as a continuous variable, are illustrated in Figures 1 and 2.

**Table 2:** Efficacy Outcomes at 90 Days Grouped by Onset-to-Treatment Time Among Modified Intention-to-Treat Population^a^

			No. of Events/Total Patients(%)		Unadjusted Analysis			Adjusted Analysis		
	Stratas	No. of Patients	Reteplase	Alteplase	Risk Ratio (95% CI)	*P* Value	*P* for Interaction	Risk Ratio (95% CI)	*P* Value	*P* for Interaction
Primary outcome										
mRS 0-1 at 90 d	Overall	1,370	549/685 (80.1)	487/685 (71.1)	1.13 (1.03-1.23)	0.006		1.13 (1.00-1.28)	0.050	
	Onset-to-treatment time^b^						0.950			0.992
	1	90	37/44 (84.1)	35/46 (76.1)	1.11 (0.84-1.46)	0.471		1.16 (0.70-1.91)	0.566	
	2	597	249/306 (81.4)	212/291 (72.9)	1.12 (1.01-1.24)	0.036		1.14 (0.97-1.35)	0.117	
	3	683	263/335 (78.5)	240/348 (69.0)	1.14 (1.02-1.27)	0.023		1.12 (0.93-1.34)	0.220	
Secondary outcome										
mRS 0-2 at 90 d	Overall	1,370	588/685 (85.8)	551/685 (80.4)	1.07 (1.01-1.13)	0.021		1.07 (0.97-1.18)	0.159	
	Onset-to-treatment time^b^						0.762			0.841
	1	90	38/44 (86.4)	38/46 (82.6)	1.05 (0.80-1.37)	0.741		1.09 (0.69-1.73)	0.713	
	2	597	266/306 (86.9)	231/291 (79.4)	1.10 (1.01-1.19)	0.032		1.11 (0.96-1.28)	0.152	
	3	683	284/335 (84.8)	282/348 (81.0)	1.05 (0.97-1.13)	0.245		1.03 (0.90-1.19)	0.626	
Median mRS at 90 d (Q1-Q3)	Overall	1,399	0 (0-1)	1 (0-2)	0.61 (0.42-0.87)^c^	0.007		0.58 (0.38-0.89)^c^	0.014	
	Onset-to-treatment time^b^						0.971			0.810
	1	91	0 (0-1)	1 (0-1)	0.58 (0.17-2.02)^c^	0.386		0.48 (0.08-3.02)^c^	0.424	
	2	612	0 (0-1)	1 (0-2)	0.64 (0.44-0.92)^c^	0.017		0.57 (0.36-0.91)^c^	0.018	
	3	696	0 (0-1)	1 (0-2)	0.60 (0.39-0.91)^c^	0.017		0.60 (0.35-1.03)^c^	0.063	
Early dramatic recovery^d^ at 24 h	Overall	1,372	398/683 (58.3)	334/689 (48.5)	1.20 (1.02-1.42)	0.028		1.19 (0.98-1.44)	0.075	
	Onset-to-treatment time^b^						0.402			0.754
	1	90	33/44 (75.0)	23/46 (50.0)	1.50 (0.98-2.31)	0.064		1.19 (0.98-1.44)	0.075	
	2	599	182/306 (59.5)	150/293 (51.2)	1.16 (0.99-1.36)	0.064		1.13 (0.91-1.40)	0.256	
	3	683	183/333 (55.0)	161/350 (46.0)	1.21 (0.99-1.48)	0.069		1.21 (0.94-1.57)	0.143	
Early dramatic recovery^d^ at 7 d	Overall	1,368	506/684 (74.0)	457/684 (66.8)	1.11 (1.01-1.21)	0.029		1.10 (0.97-1.25)	0.152	
	Onset-to-treatment time^b^						0.667			0.874
	1	90	35/44 (79.5)	29/46 (63.0)	1.26 (0.89-1.78)	0.181		1.29 (0.73-2.28)	0.369	
	2	599	235/308 (76.3)	201/291 (69.1)	1.10 (0.99-1.24)	0.089		1.08 (0.90-1.28)	0.410	
	3	679	236/332 (71.1)	227/347 (65.4)	1.09 (0.98-1.21)	0.132		1.07 (0.89-1.27)	0.476	
BI ≥95 at 90 d^e^	Overall	1,373	567/686 (82.7)	528/687 (76.9)	1.08 (1.01-1.14)	0.022		1.08 (0.97-1.19)	0.154	
	Onset-to-treatment time^b^						0.889			0.851
	1	90	38/44 (86.4)	36/46 (78.3)	1.10 (0.84-1.46)	0.483		1.23 (0.75-1.99)	0.404	
	2	598	257/306 (84.0)	225/292 (77.1)	1.09 (0.99-1.20)	0.076		1.11 (0.95-1.29)	0.175	
	3	685	272/336 (81.0)	267/349 (76.5)	1.06 (0.98-1.15)	0.166		1.05 (0.91-1.21)	0.520	

a Analyses were performed in the complete data and among the intention-to-treat population.

b Onset-to-treatment was divided into 3 groups: 1 represented the 0-90 min group, 2 represented the 91-180 min, and 3 represented the 181-270 min group.

c Shown is the common OR (reteplase vs alteplase) for a higher modified Rankin scale (mRS) score.

d Early dramatic recovery was defined as a decrease of at least 4 points in the National Institutes of Health Stroke Scale score or a score of no more than 1 point.

e Scores on the Barthel Index range from 0 to 100, with higher scores indicating better independent function.

### Safety outcomes

In the safety population, symptomatic intracranial hemorrhage within 36 hours occurred in 17 of 700 patients (2.4%) in the reteplase group and in 14 of 699 patients (2.0%) in the alteplase group (adjusted RR: 1.24; 95% CI: 0.49-3.14; *P =* 0.644). Symptomatic intracranial hemorrhage within 36 hours occurred in 1 (2.3%) of 45 patients in the reteplase group and 0 of 46 patients in the alteplase group in the OTT of 0 to 90 minutes, in 5 (1.6%) of 312 patients in the reteplase group and 7 of 300 patients in the alteplase group (adjusted RR: 0.58; 95% CI: 0.10-3.37; *P =* 0.547) in the OTT of 91 to 180 minutes, and in 11 (3.2%) of 343 patients in the reteplase group and 7 (2.0%) of 353 patients in the alteplase group (adjusted RR: 1.69; 95% CI: 0.55-5.22; *P =* 0.360) in the OTT of 181 to 270 minutes (Table 3). No significant differences were observed between the treatment and the control among different OTT groups, except that the incidence of major hemorrhage was higher in the reteplase group than in the alteplase group (4.7% vs 2.0%; adjusted RR: 2.70; 95% CI: 1.08-6.79) in the OTT of 181 to 270 minutes (Table 3).

**Table 3:** Safety Outcomes at 90 Days Grouped by Onset-to-Treatment Time^a^

			No. of Events/Total Patients (%)		Unadjusted Analysis			Adjusted Analysis		
	Stratas	No. of Patients	Reteplase	Alteplase	Risk Ratio (95% CI)	*P* Value	*P* for Interaction	Risk Ratio (95% CI)	*P* Value	*P* for Interaction
Primary safety outcome										
Symptomatic intracranial hemorrhage within 36 h^b^	Overall	1,399	17/700 (2.4)	14/699 (2.0)	1.21 (0.54-2.75)	0.644		1.24 (0.49-3.14)	0.644	
	Onset-to-treatment time^c^						0.622			0.236
	1	91	1/45 (2.2)	0/46 (0.0)	/	/		/	/	
	2	612	5/312 (1.6)	7/300 (2.3)	0.69 (0.17-2.83)	0.602		0.58 (0.10-3.37)	0.547	
	3	696	11/343 (3.2)	7/353 (2.0)	1.62 (0.62-4.21)	0.324		1.69 (0.55-5.22)	0.360	
Secondary safety outcomes										
Symptomatic intracranial hemorrhage within 7 d	Overall	1,399	17/700 (2.4)	15/699 (2.1)	1.13 (0.52-2.44)	0.753		1.14 (0.47-2.75)	0.774	
	Onset-to-treatment time^c^						0.459			0.187
	1	91	1/45 (2.2)	0/46 (0.0)	/	/		/	/	
	2	612	5/312 (1.6)	8/300 (2.7)	0.60 (0.18-2.04)	0.413		0.56 (0.11-2.92)	0.494	
	3	696	11/343 (3.2)	7/353 (2.0)	1.62 (0.62-4.21)	0.324		1.69 (0.55-5.22)	0.360	
Parenchymal hematoma 2 intracranial hemorrhage within 36 h^d^	Overall	1,399	12/700 (1.7)	10/699 (1.4)	1.20 (0.36-4.03)	0.770		1.14 (0.32-4.07)	0.834	
	Onset-to-treatment time^c^						0.424			0.498
	1	91	0/45 (0.0)	0/46 (0.0)	/	/		/	/	
	2	612	5/312 (1.6)	6/300 (2.0)	0.80 (0.18-3.51)	0.768		0.71 (0.12-4.28)	0.713	
	3	696	7/343 (2.0)	4/353 (1.1)	1.80 (0.32-10.00)	0.500		1.88 (0.35-10.21)	0.462	
Any intracranial hemorrhage within 90 d^e^	Overall	1,399	54/700 (7.7)	34/699 (4.9)	1.59 (1.00-2.51)	0.050		1.58 (0.94-2.65)	0.081	
	Onset-to-treatment time^c^						0.664			0.651
	1	91	4/45 (8.9)	2/46 (4.3)	2.04 (0.46-9.10)	0.342		3.00 (0.13-71.45)	0.491	
	2	612	21/312 (6.7)	16/300 (5.3)	1.26 (0.56-2.83)	0.571		1.43 (0.52-3.89)	0.488	
	3	696	29/343 (8.5)	16/353 (4.5)	1.87 (1.03-3.39)	0.041		1.93 (0.89-4.21)	0.097	
Major hemorrhage within 90 d	Overall	1,399	23/700 (3.3)	21/699 (3.0)	1.09 (0.65-1.85)	0.739		1.09 (0.56-2.14)	0.793	
	Onset-to-treatment time^c^						0.074			0.027
	1	91	1/45 (2.2)	1/46 (2.2)	/	/		/	/	
	2	612	6/312 (1.9)	13/300 (4.3)	0.44 (0.15-1.30)	0.138		0.41 (0.10-1.66)	0.212	
	3	696	16/343 (4.7)	7/353 (2.0)	2.35 (1.07-5.19)	0.034		2.70 (1.08-6.79)	0.034	
Clinically relevant nonmajor hemorrhage within 90 d	Overall	1,399	38/700 (5.4)	17/699 (2.4)	2.23 (1.03-4.84)	0.042		2.21 (0.94-5.20)	0.069	
	Onset-to-treatment time^c^						0.931			0.917
	1	91	2/45 (4.4)	1/46 (2.2)	/	/		/	/	
	2	612	21/312 (6.7)	10/300 (3.3)	2.03 (0.78-5.30)	0.149		2.04 (0.66-6.32)	0.215	
	3	696	15/343 (4.4)	6/353 (1.7)	2.58 (0.93-7.14)	0.069		3.03 (0.94-9.80)	0.065	
Death	Overall	1,399	30/700 (4.3)	24/699 (3.4)	1.25 (0.66-2.35)	0.492		1.21 (0.53-2.76)	0.645	
	Onset-to-treatment time^c^						0.235			0.058
	1	91	0/45 (0.0)	1/46 (2.2)	/	/		/	/	
	2	612	12/312 (3.8)	14/300 (4.7)	0.82 (0.38-1.78)	0.622		0.82 (0.22-3.00)	0.759	
	3	696	18/343 (5.2)	9/353 (2.5)	2.12 (0.85-5.31)	0.108		2.51 (0.81-7.81)	0.112	
Death within 7 d	Overall	1,399	11/700 (1.6)	11/699 (1.6)	1.00 (0.35-2.83)	0.998		0.90 (0.25-3.24)	0.873	
	Onset-to-treatment time^c^						0.529			0.129
	1	91	0/45 (0.0)	1/46 (2.2)	/	/		/	/	
	2	612	4/312 (1.3)	6/300 (2.0)	0.64 (0.16-2.54)	0.525		0.90 (0.25-3.24)	0.873	
	3	696	7/343 (2.0)	4/353 (1.1)	1.80 (0.43-7.59)	0.421		0.90 (0.25-3.24)	0.873	
Adverse event	Overall	1,399	641/700 (91.6)	576/699 (82.4)	1.11 (1.03-1.20)	0.006		1.11 (1.00-1.24)	0.057	
	Onset-to-treatment time^c^						0.125			0.429
	1	91	44/45 (97.8)	31/46 (67.4)	1.45 (1.02-2.06)	0.037		1.56 (0.90-2.70)	0.113	
	2	612	284/312 (91.0)	241/300 (80.3)	1.14 (1.04-1.24)	0.007		1.14 (0.98-1.34)	0.094	
	3	696	313/343 (91.3)	304/353 (86.1)	1.06 (0.99-1.14)	0.119		1.05 (0.92-1.20)	0.440	
Serious adverse events	Overall	1,399	105/700 (15.0)	83/699 (11.9)	1.26 (0.99-1.61)	0.057		1.28 (0.93-1.76)	0.132	
	Onset-to-treatment time^c^						0.805			0.792
	1	91	5/45 (11.1)	4/46 (8.7)	1.28 (0.36-4.58)	0.703		4.24 (0.11-160.46)	0.429	
	2	612	47/312 (15.1)	40/300 (13.3)	1.13 (0.78-1.66)	0.517		1.25 (0.73-2.12)	0.417	
	3	696	53/343 (15.5)	39/353 (11.0)	1.40 (0.92-2.12)	0.114		1.49 (0.89-2.50)	0.130	

a Analyses were performed in the safety population, which included randomly assigned patients who received reteplase or alteplase and had at least 1 post-treatment safety evaluation.

b Symptomatic intracranial hemorrhage was defined according to the European Cooperative Acute Stroke Study III definition.^2^

c Onset-to-treatment time was divided into 3 groups: 1 represented the 0-90 min group, 2 represented the 91-180 min, and 3 represented the 181-270 min group.

d Parenchymal hemorrhage type 2 was defined according to the Safe Implementation of Thrombolysis in Stroke Monitoring Study definition.^22^

e Any intracranial hemorrhage, major hemorrhage, and clinically relevant nonmassive hemorrhage were defined according to the International Society on Thrombosis and Haemostasis standard.

## Discussion

As depicted in the Central Illustration, in this subgroup analysis of the RAISE trial, we observed no significant difference in efficacy between reteplase and alteplase for achieving excellent functional outcomes at 90 days across 3 OTT groups. Furthermore, there were no significant differences in the incidence of symptomatic intracranial hemorrhage or mortality rates between the 2 treatment groups with each OTT interval. In the 181 to 270 minutes group, we observed a higher incidence of drug-related major hemorrhage in patients treated with reteplase compared with those receiving alteplase.

**Central Illustration:**
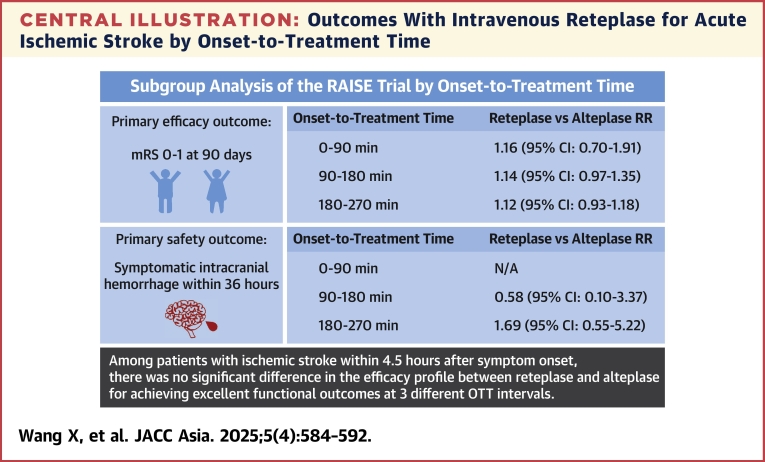
Outcomes With Intravenous Reteplase for Acute Ischemic Stroke by Onset-to-Treatment Time We aimed to delineate the associations of stroke onset-to-treatment time (OTT) on the therapeutic benefits and clinical risks with reteplase in comparison to alteplase. A total of 1,399 patients (99.1%) with OTT were included. Adjusted risk ratios (RR) of primary efficacy outcome (modified Rankin scale [mRS] score 0-1 at 90 days) were 1.16 (95% CI: 0.70-1.91) for 0 to 90 minutes group, 1.14 (95% CI: 0.97-1.35) for 91 to 180 minutes group, and 1.12 (95% CI: 0.93-1.18) for 181-270 minutes group. The primary safety outcome had no difference between reteplase and alteplase in the 3 OTT intervals. Additional studies need to be conducted to enhance the statistical power of the secondary analyses and ascertain the safety and efficacy of reteplase compared with alteplase across various OTT durations.

The benefits of intravenous thrombolysis are time-dependent. Five meta-analyses have consistently demonstrated that the earlier rt-PA is administered to stroke patients, particularly within 90 minutes, the greater the therapeutic advantage.^13^^,^^14^^,^^20^, ^21^, ^22^, ^23^ This prompt intervention during the critical “golden hour” not only accelerates blood flow restoration but also mitigates ischemic injury to brain tissue. However, previous studies investigating thrombolysis efficacy at different OTT have predominantly focused on alteplase without comparing its thrombolytic effects and risks with reteplase across various time points. The RAISE trial was the first phase 3 randomized clinical trial that comparing its efficacy and safety of reteplase vs alteplase in ischemic stroke patients within 4.5 hours after symptom onset. It revealed superior outcomes for reteplase in terms of excellent functional recovery at 90 days. The subgroup analysis revealed that reteplase showed no significant differences compared with alteplase across all OTT intervals. Additionally, a time-dependent trend was identified in the logistic regression analysis. As the onset time prolongs, the therapeutic superiority of reteplase over alteplase gradually diminishes, which might be associated with the slightly elevated risk of major bleeding in the late window of reteplase. Additional data are needed to find out the trade-off between treatment benefit and associated risks of reteplase for AIS patients presenting in the late treatment time window.

Reteplase with an extended plasma half-life allows for a more convenient double-bolus dosing regimen with fixed dosages.^4^ Reteplase has demonstrated a relatively lower affinity for fibrin in preclinical studies compared with alteplase, resulting in enhanced ability to effectively penetrate blood clots but also an increased risk of bleeding.^4^^,^^12^ This may contribute to the higher incidence of major bleeding observed early after treatment in the reteplase-treated group during the late OTT window.

### Study limitations

First, the study adopted an open-label approach. Although patients were randomly assigned and outcomes were assessed by investigators who were unaware of the group allocations, this design did not completely eliminate the potential for bias. Second, the trial was conducted mainly among the Han population with an age of <80 years in China; therefore, caution should be taken in extrapolating our findings to other populations. Furthermore, additional studies need to be conducted to enhance the statistical power of the secondary analyses and ascertain the safety and efficacy of reteplase compared with alteplase across various OTT durations.

## Conclusions

Reteplase and alteplase demonstrated equivalent efficacy profiles in achieving excellent functional outcomes at 90 days across any OTT intervals.

## Funding Support and Author Disclosures

This work was funded by China Resources Angde Biotech Pharma and Beijing Municipal Science and Technology Commission (Z221100007422050). The authors have reported that they have no relationships relevant to the contents of this paper to disclose.
