# Supplementary material for: Outcomes of Time to Treatment With Reteplase for Acute Ischemic Stroke: The RAISE Trial Subgroup
Source: JACC Asia. 2025 Feb 11;5(4):584–92. doi: 10.1016/j.jacasi.2024.12.010 (PMC12081215; doi:10.1016/j.jacasi.2024.12.010)
Supplement: Supplemental Table 1 and 2 [file mmc1.docx]

**Outcomes of Time to Treatment With Reteplase for Acute Ischemic Stroke: RAISE Trial Subgroup**

Xuechun Wang, MD ^1,2,3#^, Baoyu Feng, PhD ^2,3#^, Hong-Qiu Gu, PhD ^3^, Zixiao Li, MD ^1,2,3^, Yilong Wang, MD ^1,2,3^, Xingquan Zhao, MD ^1,2,3^, Shuya Li, MD ^1,2,3^*, Yongjun Wang, MD ^1,2,3^*

**Table S1 Baseline Characteristics of Patients in the Intention-to-Treat Population grouped by onset to treatment time.** ^*^

| **Variables** | **0-90 mins (n = 91 [6.5%])** | **91-180 mins (n = 612 [43.7%])** | **181-270 mins (n = 696 [49.7%])** | ***P* Value** |
| --- | --- | --- | --- | --- |
| Age, yr | 59.0 (54.0-66.0) | 63.5 (56.0-70.0) | 64.0 (56.0-70.0) | 0.006 |
| Age group— no. (%) |  |  |  | 0.020 |
| 18-60 yrs | 51 (56.0) | 249 (40.7) | 286 (41.1) |  |
| >60 yrs | 40 (44.0) | 363 (59.3) | 410 (58.9) |  |
| Sex— no. (%) |  |  |  | 0.005 |
| Female | 13 (14.3) | 186 (30.4) | 213 (30.6) |  |
| Male | 78 (85.7) | 426 (69.6) | 483 (69.4) |  |
| Weight, kg | 72.0 (63.0-79.0) | 68.0 (60.0-75.0) | 67.5 (60.0-75.0) | 0.040 |
| Comorbidities — no. (%) |  |  |  |  |
| Hypertension | 61 (67.0) | 460 (75.2) | 525 (75.4) | 0.210 |
| Diabetes | 19 (20.9) | 150 (24.5) | 183 (26.3) | 0.470 |
| Hyperlipidaemia | 53 (58.2) | 245 (40.0) | 273 (39.2) | 0.002 |
| Coronary heart disease | 29 (31.9) | 160 (26.1) | 169 (24.3) | 0.270 |
| Arrhythmia^‡^ | 13 (14.3) | 90 (14.7) | 100 (14.4) | 0.980 |
| mRS score before stroke^¶^— no. (%) |  |  |  | 0.950 |
| 0 | 84 (92.3) | 568 (92.8) | 648 (93.1) |  |
| 1 or 2 ^‖^ | 7 (7.7) | 44 (7.2) | 48 (6.9) |  |
| NIHSS score at admission§ | 6.0 (5.0-9.0) | 6.0 (5.0-9.0) | 6.0 (5.0-8.0) | 0.110 |
| NIHSS score group at admission^§^— no. (%) |  |  |  | 0.040 |
| 4-7 | 55 (60.4) | 388 (63.4) | 482 (69.3) |  |
| >7 | 36 (39.6) | 224 (36.6) | 214 (30.7) |  |
| Symptom onset to treatment time | 76.0 (62.0-87.0) | 141.0 (119.0-162.0) | 222.0 (200.0-244.0) | <0.001 |
| Door to treatment time | 36.0 (29.0-49.0) | 57.0 (38.0-76.5) | 67.0 (43.5-95.0) | <0.001 |
| Bridging thrombectomy— no. (%) | 2 (2.2) | 28 (4.6) | 15 (2.2) | 0.040 |

Note: * Data were not applicable for 13 patients (7 in the reteplase group and 6 in the alteplase group) because they did not receive the assigned treatment.

‡ Arrhythmia includes sinus bradycardia, sinus tachycardia, atrial flutter, atrial fibrillation, premature beats, supraventricular tachycardia, ventricular tachycardia, bundle-branch block, atrioventricular block, and idioventricular rhythm.

¶ Scores on the modified Rankin scale range from 0 (no neurologic deficit, no symptoms, or completely recovered) to 6 (death).

‖ One patient in each group had a modified Rankin scale score of 2.

§ Scores on the National Institutes of Health Stroke Scale (NIHSS) range from 0 to 42, with higher scores indicating more severe stroke.

| **Table S2** **Subgroup Analysis of Efficacy outcome mRS 0-1 at 90 days.** | | | | | | | | | | | | |
| --- | --- | --- | --- | --- | --- | --- | --- | --- | --- | --- | --- | --- |
|  | | | **No. of events/Total patients(%)** | | **Unadjusted Analysis** | | |  | **Adjusted Analysis** | | |  |
| **Outcome** | **Stratas** | **No.of patients** | **Reteplase** | **Alteplase** | **Risk Ratio (95% CI)** | **P Value** | **P for Interaction** |  | **Risk Ratio (95% CI)** | **P Value** | **P for Interaction** |  |
| OTT within 90 min | Overall | 1370 | 549/685 (80.1) | 487/685 (71.1) | 1.13 (1.03-1.23) | 0.006 |  |  | 1.13 (1.00-1.28) | 0.050 |  |  |
|  | Age |  |  |  |  |  | 0.328 |  |  |  | 0.387 |  |
|  | 18 ~ 60 years | 50 | 19/24 (79.2) | 22/26 (84.6) | 0.94 (0.60-1.46) | 0.761 |  |  | 0.99 (0.46-2.16) | 0.989 |  |  |
|  | > 60 years | 40 | 18/20 (90.0) | 13/20 (65.0) | 1.38 (0.71-2.69) | 0.323 |  |  | 2.17 (0.27-17.58) | 0.445 |  |  |
|  | Sex |  |  |  |  |  | 0.841 |  |  |  | 0.984 |  |
|  | Female | 13 | 6/7 (85.7) | 5/6 (83.3) | 1.03 (0.35-3.04) | 0.951 |  |  | (-) |  |  |  |
|  | Male | 77 | 31/37 (83.8) | 30/40 (75.0) | 1.12 (0.82-1.51) | 0.469 |  |  | 1.19 (0.67-2.13) | 0.539 |  |  |
|  | Hypertension |  |  |  |  |  | 0.959 |  |  |  | 0.855 |  |
|  | No | 30 | 14/16 (87.5) | 11/14 (78.6) | 1.11 (0.59-2.12) | 0.729 |  |  | 2.04 (0.28-15.20) | 0.435 |  |  |
|  | Yes | 60 | 23/28 (82.1) | 24/32 (75.0) | 1.10 (0.75-1.61) | 0.634 |  |  | 1.11 (0.54-2.30) | 0.768 |  |  |
|  | Diabetes |  |  |  |  |  | 0.988 |  |  |  | 0.773 |  |
|  | No | 71 | 29/35 (82.9) | 27/36 (75.0) | 1.10 (0.80-1.53) | 0.543 |  |  | 1.23 (0.66-2.29) | 0.504 |  |  |
|  | Yes | 19 | 8/9 (88.9) | 8/10 (80.0) | 1.11 (0.43-2.87) | 0.805 |  |  | 1.00 (-) |  |  |  |
|  | Hyperlipidemia |  |  |  |  |  | 0.648 |  |  |  | 0.664 |  |
|  | No | 37 | 15/18 (83.3) | 13/19 (68.4) | 1.22 (0.64-2.30) | 0.527 |  |  | 1.78 (0.31-10.30) | 0.492 |  |  |
|  | Yes | 53 | 22/26 (84.6) | 22/27 (81.5) | 1.04 (0.68-1.58) | 0.856 |  |  | 1.03 (0.57-1.85) | 0.922 |  |  |
|  | Coronary heart disease |  |  |  |  |  | 0.610 |  |  |  | 0.793 |  |
|  | No | 62 | 25/31 (80.6) | 24/31 (77.4) | 1.04 (0.69-1.57) | 0.842 |  |  | 1.09 (0.58-2.05) | 0.785 |  |  |
|  | Yes | 28 | 12/13 (92.3) | 11/15 (73.3) | 1.26 (0.63-2.51) | 0.487 |  |  | 0.45 (0.00-366.03) | 0.770 |  |  |
| OTT between 91 and 180 min | Age |  |  |  |  |  | 0.150 |  |  |  | 0.379 |  |
|  | 18 ~ 60 years | 240 | 96/110 (87.3) | 109/130 (83.8) | 1.04 (0.90-1.20) | 0.586 |  |  | 1.04 (0.80-1.36) | 0.758 |  |  |
|  | > 60 years | 357 | 153/196 (78.1) | 103/161 (64.0) | 1.22 (1.05-1.42) | 0.011 |  |  | 1.21 (0.96-1.53) | 0.114 |  |  |
|  | Sex |  |  |  |  |  | 0.587 |  |  |  | 0.772 |  |
|  | Female | 180 | 68/88 (77.3) | 61/92 (66.3) | 1.17 (0.94-1.44) | 0.155 |  |  | 1.24 (0.87-1.76) | 0.241 |  |  |
|  | Male | 417 | 181/218 (83.0) | 151/199 (75.9) | 1.09 (0.98-1.22) | 0.106 |  |  | 1.12 (0.92-1.37) | 0.255 |  |  |
|  | Hypertension |  |  |  |  |  | 0.071 |  |  |  | 0.325 |  |
|  | No | 146 | 70/83 (84.3) | 55/63 (87.3) | 0.97 (0.80-1.16) | 0.714 |  |  | 1.04 (0.72-1.51) | 0.834 |  |  |
|  | Yes | 451 | 179/223 (80.3) | 157/228 (68.9) | 1.17 (1.04-1.31) | 0.008 |  |  | 1.21 (1.01-1.45) | 0.041 |  |  |
|  | Diabetes |  |  |  |  |  | 0.081 |  |  |  | 0.310 |  |
|  | No | 452 | 184/229 (80.3) | 170/223 (76.2) | 1.05 (0.93-1.19) | 0.402 |  |  | 1.11 (0.91-1.36) | 0.309 |  |  |
|  | Yes | 145 | 65/77 (84.4) | 42/68 (61.8) | 1.37 (1.07-1.74) | 0.012 |  |  | 1.23 (0.81-1.88) | 0.327 |  |  |
|  | Hyperlipidemia |  |  |  |  |  | 0.625 |  |  |  | 0.501 |  |
|  | No | 359 | 147/186 (79.0) | 125/173 (72.3) | 1.09 (0.95-1.26) | 0.216 |  |  | 1.07 (-) | <.001 |  |  |
|  | Yes | 238 | 102/120 (85.0) | 87/118 (73.7) | 1.15 (0.99-1.34) | 0.059 |  |  | 1.26 (0.95-1.65) | 0.104 |  |  |
|  | Coronary heart disease |  |  |  |  |  | 0.740 |  |  |  | 0.943 |  |
|  | No | 442 | 187/225 (83.1) | 163/217 (75.1) | 1.11 (0.98-1.25) | 0.100 |  |  | 1.13 (0.92-1.38) | 0.256 |  |  |
|  | Yes | 155 | 62/81 (76.5) | 49/74 (66.2) | 1.16 (0.93-1.44) | 0.197 |  |  | 1.17 (0.80-1.72) | 0.419 |  |  |
| OTT over 180 min | Age |  |  |  |  |  | 0.702 |  |  |  | 0.832 |  |
|  | 18 ~ 60 years | 278 | 123/145 (84.8) | 102/133 (76.7) | 1.11 (0.96-1.27) | 0.150 |  |  | 1.11 (0.87-1.42) | 0.386 |  |  |
|  | > 60 years | 405 | 140/190 (73.7) | 138/215 (64.2) | 1.15 (0.99-1.33) | 0.065 |  |  | 1.19 (0.94-1.50) | 0.141 |  |  |
|  | Sex |  |  |  |  |  | 0.249 |  |  |  | 0.604 |  |
|  | F | 211 | 71/96 (74.0) | 82/115 (71.3) | 1.04 (0.83-1.30) | 0.750 |  |  | 1.01 (0.71-1.43) | 0.958 |  |  |
|  | M | 472 | 192/239 (80.3) | 158/233 (67.8) | 1.18 (1.06-1.32) | 0.002 |  |  | 1.13 (0.93-1.38) | 0.228 |  |  |
|  | Hypertension |  |  |  |  |  | 0.349 |  |  |  | 0.924 |  |
|  | No | 168 | 64/74 (86.5) | 76/94 (80.9) | 1.07 (0.92-1.25) | 0.395 |  |  | 1.16 (0.82-1.64) | 0.391 |  |  |
|  | Yes | 515 | 199/261 (76.2) | 164/254 (64.6) | 1.18 (1.02-1.36) | 0.024 |  |  | 1.09 (0.88-1.36) | 0.422 |  |  |
|  | Diabetes |  |  |  |  |  | 0.523 |  |  |  | 0.628 |  |
|  | No | 501 | 194/237 (81.9) | 185/264 (70.1) | 1.17 (1.03-1.32) | 0.015 |  |  | 1.16 (0.95-1.40) | 0.140 |  |  |
|  | Yes | 182 | 69/98 (70.4) | 55/84 (65.5) | 1.08 (0.86-1.35) | 0.525 |  |  | 1.00 (0.66-1.51) | 0.989 |  |  |
|  | Hyperlipidemia |  |  |  |  |  | 0.552 |  |  |  | 0.824 |  |
|  | No | 415 | 166/209 (79.4) | 140/206 (68.0) | 1.17 (0.99-1.39) | 0.074 |  |  | 1.12 (0.86-1.46) | 0.414 |  |  |
|  | Yes | 268 | 97/126 (77.0) | 100/142 (70.4) | 1.09 (0.95-1.26) | 0.228 |  |  | 1.11 (0.86-1.45) | 0.414 |  |  |
|  | Coronary heart disease |  |  |  |  |  | 0.915 |  |  |  | 0.959 |  |
|  | No | 517 | 209/259 (80.7) | 183/258 (70.9) | 1.14 (1.01-1.28) | 0.028 |  |  | 1.13 (0.92-1.38) | 0.245 |  |  |
|  | Yes | 166 | 54/76 (71.1) | 57/90 (63.3) | 1.12 (0.87-1.44) | 0.364 |  |  | 1.12 (1.12-1.12) | <.001 |  |  |

Adjusted variables: age, weight, NIHSS score at admission, mRS score, sex, hypertension, diabetes, hyperlipidemia, coronary heart disease, and arrhythmia. In the specific subgroup, the stratified factor was not adjusted.
